# Utility of Transpapillary Biopsy and Endoscopic Ultrasound-Guided Tissue Acquisition for Comprehensive Genome Profiling of Unresectable Biliary Tract Cancer

**DOI:** 10.3390/cancers16162819

**Published:** 2024-08-10

**Authors:** Soma Fukuda, Susumu Hijioka, Yoshikuni Nagashio, Daiki Yamashige, Daiki Agarie, Yuya Hagiwara, Kohei Okamoto, Shin Yagi, Yasuhiro Komori, Masaru Kuwada, Yuta Maruki, Chigusa Morizane, Hideki Ueno, Nobuyoshi Hiraoka, Kiichiro Tsuchiya, Takuji Okusaka

**Affiliations:** 1 Department of Hepatobiliary and Pancreatic Oncology, National Cancer Center Hospital, 5-1-1 Tsukiji, Chuo-ku, Tokyo 104-0045, Japan; sofukud@ncc.go.jp (S.F.); yonagash@ncc.go.jp (Y.N.); dyamashi@ncc.go.jp (D.Y.); dagarie@ncc.go.jp (D.A.); yuhagiw2@ncc.go.jp (Y.H.); kookamot@ncc.go.jp (K.O.); shyag@ncc.go.jp (S.Y.); yakomor@ncc.go.jp (Y.K.); mkuwada@ncc.go.jp (M.K.); ymaruki@ncc.go.jp (Y.M.); cmorizan@ncc.go.jp (C.M.); hiueno@ncc.go.jp (H.U.); tokusaka@ncc.go.jp (T.O.); 2 Department of Gastroenterology, Institute of Medicine, University of Tsukuba, 1-1-1 Tennodai, Tsukuba 305-8575, Japan; kii.gast@md.tsukuba.ac.jp; 3 Department of Diagnostic Pathology, National Cancer Center Hospital, 5-1-1 Tsukiji, Chuo-ku, Tokyo 104-0045, Japan; nhiraoka@ncc.go.jp

**Keywords:** comprehensive genome profiling, biliary tract cancer, cholangiocarcinoma, transpapillary biopsy, endoscopic ultrasound-guided tissue acquisition, OncoGuide NCC Oncopanel System, FoundationOne CDx

## Abstract

**Simple Summary:**

There are only a few reports on comprehensive genome profiling (CGP) analysis using transpapillary biopsy (TPB) and endoscopic ultrasound-guided tissue acquisition (EUS-TA) specimens from primary lesions in biliary tract cancers (BTCs). This study compared TPB and EUS-TA for their suitability in CGP analysis of unresectable BTC. We used the OncoGuide NCC Oncopanel System (NCCOP) and FoundationOne CDx (F1CDx) as criteria for CGP analysis. Among 78 patients (35 TPB, 43 EUS-TA) EUS-TA showed significantly higher suitability for NCCOP analysis (34.9% vs. 8.6%, *p* = 0.007), whereas F1CDx analysis suitability was 0% in both groups. EUS-TA was identified as an independent factor contributing to NCCOP analysis suitability. EUS-TA specimens were more suitable for analysis in mass lesions (43.8% vs. 9.1%, *p* = 0.065) and less suitable in perihilar cholangiocarcinoma (0% vs. 41.7%, *p* = 0.077), whereas TPB showed potential for analysis in papillary-type lesions and peroral cholangioscopy-assisted biopsies. Overall, EUS-TA is preferable for CGP analysis in unresectable BTC, with TPB potentially serving as a complementary method.

**Abstract:**

Tissue sampling in biliary tract cancer (BTC) is generally performed through transpapillary biopsy (TPB) or endoscopic ultrasound-guided tissue acquisition (EUS-TA). For the first time, we compared the suitability of specimens obtained using TPB and EUS-TA to determine the optimal tissue-sampling method for comprehensive genome profiling (CGP) analysis in patients with unresectable BTC (UR-BTC). Pathology precheck criteria for CGP analysis comprised the OncoGuide NCC Oncopanel System (NCCOP) and FoundationOne CDx (F1CDx). Seventy-eight patients with UR-BTC (35 TPB and 43 EUS-TA) were included. The NCCOP analysis suitability achievement rate was higher in EUS-TA specimens than in TPB specimens (34.9% vs. 8.6%, *p* = 0.007), whereas that of F1CDx was 0% in both groups. EUS-TA was identified as an independent factor that contributed to the suitability of the NCCOP analysis. The suitability of the NCCOP analysis of EUS-TA specimens showed a tendency to be higher for mass lesions (43.8% vs. 9.1%, *p* = 0.065), especially for target size ≥ 18.5 mm, and lower for perihilar cholangiocarcinoma (0% vs. 41.7%, *p* = 0.077). In TPB, papillary-type lesions (66.7% vs. 3.2%, *p* = 0.016) and peroral cholangioscopy-assisted biopsies (50.0% vs. 3.3%, *p* = 0.029) showed better potential for successful NCCOP analysis. EUS-TA is suitable for NCCOP analysis in UR-BTC and may be partially complemented by TPB.

## 1. Introduction

Advanced biliary tract cancer (BTC) has a poor prognosis, with a 5-year survival rate of 7%–20% [1]. Recent advances in comprehensive genome profiling (CGP) using tissue- and blood-derived DNA have raised hopes for mutation-specific therapy in patients with unresectable BTC (UR-BTC), where treatment options are limited [2,3]. CGP can be performed using tumor tissue or liquid biopsy sampling, but liquid biopsy using blood samples can result in false-negative results due to factors such as sampling time and tumor volume [4]; in particular, the detection of fusion genes has been reported to be poor [5,6]. The *FGFR2* fusion gene is one of the most common and important therapeutic targets in BTC [7], and there are many reports that an improved prognosis in patients with UR-BTC has been achieved with treatment with *FGFR2* inhibitors [8,9,10]. Therefore, when performing CGP in UR-BTC, it is advisable to obtain tissue samples whenever possible.

CGP requires a large number of tumor tissue samples for next-generation sequencing (NGS), but there is no established method of tissue sampling suitable for CGP in UR-BTC. Although no reports of actual CGP using transpapillary biopsy (TPB) specimens are available, it is often the first choice for tissue sampling in patients with BTC, but due to the low sensitivity of 49%–67% for the diagnosis of malignancy and the small size of specimens [11,12], the clinical use of TPB specimens for CGP has not progressed.

Recently, endoscopic ultrasound-guided tissue acquisition (EUS-TA) for BTC has been shown to have a higher diagnostic performance than TPB, with a sensitivity of 73%–96% and specificity of 99%–100%, and is safe with 0%–0.5% adverse events reported [11,12,13,14,15]. The next step is to investigate whether EUS-TA for BTC is useful as a sampling method to obtain genomic data, as in pancreatic cancer.

Regarding CGP using EUS-TA, many reports have shown its usefulness in pancreatic cancer with CGP analysis success rates of 39.2–100% [16,17,18,19,20,21,22,23,24], but only two studies have been published for patients with BTC [25,26]. Furthermore, both articles included many tissue samples from lymph nodes and liver metastases, and the success rate of CGP analysis of EUS-TA for primary tumors of BTC is not yet clear. Therefore, our objective was to evaluate the achievement rate of the CGP analysis tissue criteria in TPB and EUS-TA specimens obtained from primary lesions from patients with UR-BTC. 

## 2. Materials and Methods

### 2.1. Study Population

This single-center retrospective study was conducted at the National Cancer Center Hospital (Tokyo, Japan) from October 2017 to February 2024. Consecutive patients with UR-BTC who were diagnosed with malignancy from primary lesions by TPB under endoscopic retrograde cholangiopancreatography or EUS-TA were included. BTCs included intrahepatic cholangiocarcinoma (iCCA), perihilar cholangiocarcinoma (pCCA), distal cholangiocarcinoma (dCCA), gallbladder cancer (GBC), and ampullary cancer (AC). Patients with a pathological diagnosis based solely on cytology, lymph node biopsy, or distant metastases alone, or post-operative recurrent cases of BTC, were excluded. Cases in which malignancy was obtained by both TPB and EUS-TA were included only for the first tissue sample. Information on baseline characteristics, the TPB and EUS-TA technique, pathological data, and actual CGP results were collected retrospectively from medical records. The study was approved by the Institutional Review Board (IRB) of the National Cancer Center (study no.: 2018-149). The IRB waived the requirement for informed consent, and all patient information was deidentified.

### 2.2. Endoscopic Procedures

In TPB, standard biopsy forceps for the upper gastrointestinal tract (EndoJaw FB-211K Alligator Jaw; Olympus, Tokyo, Japan), one-sided opening-cup biopsy forceps (FB-46Q-1; Olympus), or peroral cholangioscopy (POCS) compatible biopsy forceps (SpyBite, SpyBite Max; Boston Scientific, Marlborough, MA, USA) were used. At least two biopsy specimens of biliary stricture were routinely taken, and the number of tissue samples was increased if the tissue obtained was small or insufficient. Each specimen was soaked in a bottle containing a 10% formalin solution, and all specimens were submitted for pathological evaluation. 

A 22-gauge fine-needle biopsy (FNB) needle was used for EUS-TA, and a 22-gauge fine-needle aspiration (FNA) needle was used if the FNB needle was difficult to puncture with, as when the FNB needle does not penetrate the bowel wall or target lesion well or deviates significantly from the expected puncture line. The use of a 19-gauge FNB needle was considered when samples were collected for CGP. For the FNB needle, Franseen needles (Acquire, Boston Scientific, or SonoTip TopGain, Medico’s Hirata, Osaka, Japan) or a Fork-Tip needle (SharkCore, Medtronic, Dublin, Ireland) were used. For the FNA needle, a Lancet needle (EZ Shot 3 Plus; Olympus) was used. The aspiration method during puncture involved negative pressure (20 mL) suction for the FNA needle and a slow-pull technique (sampling performed with simultaneous minimal negative pressure provided by retracting the needle stylet slowly) [27] for the FNB needle. The number of strokes was generally approximately 20. In all cases, rapid on-site evaluation (ROSE) with Diff-Quik staining was performed on a portion of the specimen. In many cases, two or three additional punctures were performed after atypical or malignant cells were confirmed with ROSE. Each collected specimen was soaked in a bottle containing a 10% formalin solution. The red component, which contains blood, was not separated from the white component, and all samples obtained were submitted for pathological evaluation.

### 2.3. Endpoints

The primary endpoint was the proportion of patients who met the CGP analysis suitability criteria based on a pathologist’s precheck of specimens collected from primary tumors in patients with UR-BTC. The secondary endpoints were tumor cellularity and tissue size, the proportion of successful CGP analyses performed in practice, and the rate of procedure-related adverse events. These endpoints were compared between the TPB and EUS-TA groups.

### 2.4. Outcome Measures

As criteria for CGP testing, the OncoGuide NCC Oncopanel System (NCCOP; Sysmex, Hyogo, Japan) and FoundationOne CDx (F1CDx; Foundation Medicine, Cambridge, MA, USA), which are currently widely used and reimbursed by the national insurance system in Japan, were applied. The NCCOP analyzes both tumor and nontumor DNA (by peripheral blood), therefore, germline mutations will be identified. Based on previous reports, NCCOP analysis suitability criteria were defined as tumor cellularity ≥ 20% and tissue area ≥ 4 mm^2^ [28,29]. Meeting these tissue criteria fulfilled the 200 ng of DNA required for the NCCOP analysis. The suitability criteria for F1CDx were defined as tumor cellularity ≥ 20% and tissue area ≥ 25 mm^2^ [30]. In the precheck of the tissue samples, an experienced pathologist (N.H.) assessed whether the specimens were suitable for CGP analysis based on these criteria.

Hematoxylin- and eosin-stained slides judged to be most suitable for CGP analysis were used for evaluation. For patients who actually underwent CGP analysis, tumor cellularity, tissue area, DNA content, and detected genetic mutations as described in the CGP analysis report were used for evaluation.

### 2.5. Definitions

The primary site of UR-BTC and T category were classified according to the Union for International Cancer Control TNM classification (8th edition) by contrast-enhanced computed tomography (CECT). The form of the primary tumor was classified as a mass lesion or thickened wall based on CECT and EUS. The macroscopic type of primary tumor was classified as papillary, nodular, or flat type for pCCA, dCCA, GBC, and AC, and as mass-forming, periductal-infiltrating, and intraductal-growth type for iCCA, based on imaging findings such as CECT, EUS, and cholangiography [31] (Figure S1). The target size was measured using CECT (axial or coronal images) within 28 days before tissue collection in the TPB group and EUS in the EUS-TA group, with mass lesions measured as the largest diameter of the mass and the thickened walls as the largest thickness of the wall. Procedural adverse events were evaluated using the American Society for Gastrointestinal Endoscopy Lexicon [32].

### 2.6. Statistical Analysis

Continuous variables are expressed as medians (interquartile ranges [IQRs]), and categorical variables as numbers (percentages). Statistical comparisons were made using the χ^2^ test or Fisher’s exact test for categorical variables and the Mann–Whitney U test for continuous variables. In univariate and multivariate analyses to evaluate the contributing factors for CGP analysis suitability, binary logistic regression was used to calculate the odds ratio (OR) with a 95% confidence interval (CI). Factors with *p*-values of <0.10 on univariate analysis were further evaluated by multivariate analysis. The area under the receiver operating characteristic (ROC) curve was calculated to determine the optimal target size of the mass lesions for CGP in the EUS-TA group. *p*-values of <0.05 were considered statistically significant. All statistical analyses were performed using SPSS Statistics (version 27.0; IBM Corp., Armonk, New York, NY, USA).

## 3. Results

### 3.1. Patient Characteristics

**Figure 1:**
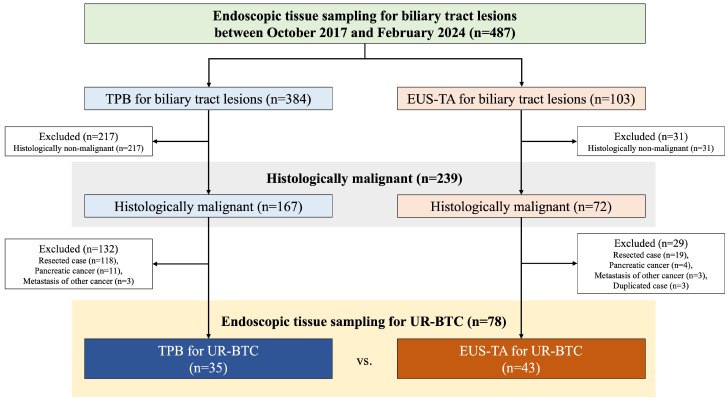
Flow diagram of the study. TPB, transpapillary biopsy; EUS-TA, endoscopic ultrasound-guided tissue acquisition; UR-BTC, unresectable biliary tract cancer.

Of a total of 487 patients (384 TPB, 103 EUS-TA) who underwent endoscopic tissue sampling for biliary tract lesions during the study period, 239 patients (167 TPB, 72 EUS-TA) were diagnosed as having malignant tumors (Figure 1). Of these, 161 patients (132 TPB, 29 EUS-TA) were excluded for the following reasons: (i) resected cases, (ii) pancreatic cancer, (iii) metastasis of other cancers, and (iv) duplicated cases. Therefore, 78 patients with UR-BTC (35 TPB, 43 EUS-TA) were included in the study.

Patient characteristics are summarized in Table 1. The primary tumor site was significantly more prevalent as pCCA in the TPB group than in the EUS-TA group (57.1% vs. 16.3%, *p* < 0.001), and significantly less as iCCA (2.9% vs. 44.2%, *p* < 0.001). The form of the primary tumor was not significantly different between the TPB and EUS-TA groups, with 24 (68.6%) and 32 (74.4%) mass lesions, whereas 11 (31.4%) and 11 (25.6%) presented thickened walls, respectively (*p* = 0.619). There were also significantly fewer cases with distant metastases in the TPB group (51.4% vs. 76.7%, *p* = 0.031). There was no significant difference in the proportion of macroscopic type of primary tumor between the two groups (Table S1).

### 3.2. Procedure Details

Details of the procedures are presented in Table 2. The most common biopsy device used in the TPB group was POCS-compatible biopsy forceps, which were used in 20 cases (57.1%), of which 4 cases (11.4%) were biopsied using POCS. The type of needle used in the EUS-TA group was an FNB needle in 32 cases (74.4%) and an FNA needle in 11 cases (25.6%). The needle size was 22 gauge in 35 cases (81.4%) and 19 gauge in 8 cases (18.6%). In 25 cases (58.1%), the puncture was performed transduodenally. The aspiration method after puncture was used in 30 cases (69.8%) by slow-pull and in 13 cases (30.2%) by suction (20 mL). The median target size was not significantly different between the TPB and EUS-TA groups, with a mass lesion of 24.5 mm (IQR 18.9–30.4) and 34.3 mm (IQR 20.0–46.9) (*p* = 0.122), whereas the thickened wall was 6.0 mm (IQR 4.3–8.0) and 7.3 mm (IQR 7.0–9.4), respectively (*p* = 0.263).

### 3.3. Outcomes

**Figure 2:**
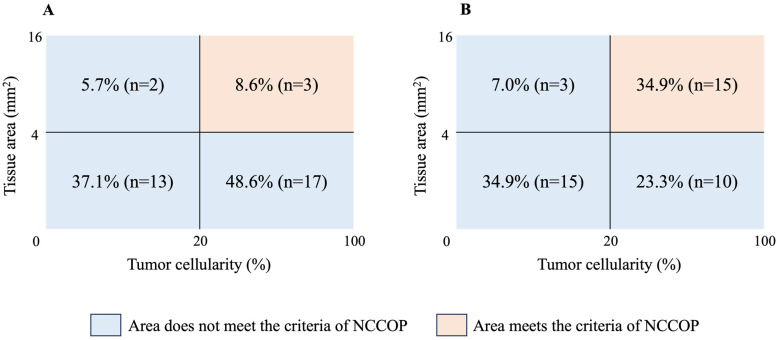
Distribution for each suitability criterion of OncoGuide NCC Oncopanel System (NCCOP) analysis. (**A**) Transpapillary biopsy (TPB) group (*n* = 35); (**B**) Endoscopic ultrasound-guided tissue acquisition (EUS-TA) group (*n* = 43).

Table 3 and Figure 2 show the proportion of patients who met the suitability of the CGP analysis. The proportion of patients meeting tumor cellularity ≥20% was 57.1% (20/35) and 58.1% (25/43) in the TPB and EUS-TA groups, respectively (*p* = 1.000). The proportion of patients with tissue area ≥ 4 mm^2^ was 14.3% (5/35) and 41.9% (18/43) in the TPB and EUS-TA groups, respectively, which was significantly higher in the EUS-TA group than in the TPB group (*p* = 0.012). The results show that the achievement rate of NCCOP analysis suitability was 8.6% (3/35) and 34.9% (15/43) in the TPB and EUS-TA groups, respectively, and was significantly higher in the EUS-TA group than in the TPB group (*p* = 0.007). The NCCOP analysis did not meet the criteria for the following reasons in the TPB and EUS-TA groups, respectively: insufficient tumor cellularity at only 5.7% (2/35) and 7.0% (3/43) (*p* = 1.000), insufficient tissue area at only 48.6% (17/35) and 23.3% (10/43) (*p* = 0.031), and combined insufficiency of tumor cellularity and tissue area at 37.1% (13/35) and 34.9% (15/43) (*p* = 1.000). Conversely, no samples (tissue area ≥ 25 mm^2^) suitable for F1CDx analysis were obtained in either group.

The percentage of samples collected that met the NCCOP analysis suitability by primary site in both groups is shown in Table S2: iCCA 0% (0/1) vs. 36.8% (7/19) (*p* = 1.000) in the TPB and EUS-TA groups, respectively; pCCA 10.0% (2/20) vs. 0% (0/7) (*p* = 1.000), dCCA 25.0% (1/4) vs. 33.3% (1/3) (*p* = 1.000), GBC 0% (0/5) vs. 41.7% (5/12) (*p* = 0.245), and AC 0% (0/5) vs. 100% (2/2) (*p* = 0.048).

Table 4 shows the results of the analysis of factors associated with the suitability of the NCCOP analysis for UR-BTC. In univariate analysis to evaluate the suitability of the NCCOP analysis, the EUS-TA group suitability was significantly better than that of the TPB group (34.9% vs. 8.6%, *p* = 0.007), pCCA was significantly worse (7.4% vs. 29.0%, *p* = 0.023), and mass lesions tended to be better than thickened walls (28.6% vs. 9.1%, *p* = 0.080). In the multivariate analysis, only EUS-TA (adjusted OR 5.32, 95% CI 1.31–21.6; *p* = 0.019) was extracted as an independent factor associated with achieving the suitability of the NCCOP analysis.

Subgroup analyses of factors associated with NCCOP analysis suitability in both groups are shown in Table 5 and Table 6, respectively. In the TPB group, the achievement rate of NCCOP analysis suitability was significantly higher for papillary-type lesions (66.7% vs. 3.2%, *p* = 0.016) and for POCS-assisted biopsies (50.0% vs. 3.3%, *p* = 0.029). In terms of the rate for the EUS-TA group, the achievement of NCCOP analysis suitability tended to be lower for pCCA (0% vs. 41.7%, *p* = 0.077) and higher for mass lesions (43.8% vs. 9.1%, *p* = 0.065). No significant differences were found in the achievement rate of NCCOP analysis suitability by needle type (FNA or FNB), needle size (22 gauge or 19 gauge), or aspiration method (suction or slow-pull). Using ROC curves, the appropriate cutoff value for the target mass-lesion size to predict the suitability of the NCCOP analysis was established at 18.5 mm in the EUS-TA group. Mass lesions with target size ≥18.5 mm tended to have higher NCCOP analysis suitability (51.9% vs. 0%, *p* = 0.053).

### 3.4. Actual CGP Analysis

Of the specimens that met the NCCOP analysis suitability in the UR-BTC group, 0% (0/3) and 26.7% (4/15) were actually submitted to NCCOP analysis in the TPB and EUS-TA groups, respectively. The actual success rate of NCCOP analysis in the EUS-TA group was 100% (4/4), and the amount of DNA in all specimens exceeded 200 ng (Table S3). Four cases consisted of one iCCA, two GBC, and one AC case, all using a 22-gauge FNB needle. Mutations in *IDH1* and *TP53* were detected in iCCA (Case 1); *GNAQ*, *SMAD4,* and *TP53* were detected in GBC (Case 2); *ATM* was detected in GBC (Case 3); and *ARID2*, *RB1*, and *TP53* were detected in AC (Case 4).

### 3.5. Procedural Adverse Events

The rate of procedure-related adverse events was 17.1% (6/35) in the TPB group: mild pancreatitis in 8.6% (3/35), severe pancreatitis in 2.9% (1/35), mild cholangitis in 2.9% (1/35), and mild fever in 2.9% (1/35); however, the rate of adverse event was 2.3% (1/43) in the EUS-TA group (only mild fever), with significantly fewer adverse events in the EUS-TA group (*p* = 0.041). No case of biliary peritonitis or needle-tract seeding was observed in the EUS-TA group during the median observation period of 169 days (range 12–1052 days).

## 4. Discussion

In this study, endoscopic tissue collection by EUS-TA showed significantly better NCCOP analysis suitability than did TPB from the primary lesion for patients with UR-BTC (34.9% vs. 8.6%, *p* = 0.007). Moreover, EUS-TA was determined to be an independent factor that contributed to NCCOP analysis suitability. The NCCOP analysis suitability of EUS-TA specimens tended to be higher for mass lesions, especially for target size ≥ 18.5 mm, and lower for pCCA lesions. Although the general suitability of the NCCOP analysis of TPB was low, papillary-type lesions and POCS-assisted biopsies were considered to have the potential for more successful NCCOP analysis. Furthermore, a small number of cases in pCCA met NCCOP analysis suitability by TPB. The results suggest that the use of EUS-TA and TPB according to the primary site may contribute to NCCOP analysis suitability. To the best of our knowledge, this is the first study to compare the CGP analysis suitability of TPB and EUS-TA for primary lesions of BTC.

Lamarca et al. reported the failure of NGS in 26.8% of tissue samples (including surgical specimens) submitted for CGP and obtained from patients with BTC, thus, appropriate tissue sampling for CGP in BTC remains a major challenge [33]. A previous report of gene-panel testing using EUS-TA specimens from 21 patients with BTC reported that a unique gene panel (50 genes) was successfully analyzed, and oncogene mutations were identified in 95.2% (20/21) of patients [25]. However, 42.9% (9/21) of the puncture targets in this study were lymph nodes, and the number of cancer genes analyzed was much lower than that of CGPs approved for insurance in Japan, which may have led to good results. In a recent single-center retrospective study by Yanaidani et al. using three CGPs— F1CDx, NCCOP, and MSK-IMPACT (Memorial Sloan Kettering-Integrated Mutation Profiling of Actionable Cancer Targets)—the success rate for CGP analysis of BTC was reported to be 93.8% (30/32) for surgical specimens, 76.5% (39/51) for EUS-TA specimens, and 64.7% (11/17) for percutaneous liver biopsy specimens, suggesting the utility of EUS-TA for CGP analysis [26]. However, in this study, only specimens that had already passed the pathologist’s precheck were submitted and evaluated for CGP testing, and the success rate for the entire case population was likely much lower. This study also included many tissue samples from lymph nodes and liver metastases, and the CGP feasibility of EUS-TA for the primary lesion of BTC itself is not clear.

In this study, 34.9% of EUS-TA for primary UR-BTC lesions met the suitability criteria of the NCCOP analysis, suggesting that mass lesions, especially those with target size ≥ 18.5 mm, may be suitable. Conversely, none of the seven EUS-TA cases obtained from patients with pCCA met NCCOP analysis suitability. Previous reports on the diagnostic performance of EUS-TA for BTC have also reported a lower positive diagnosis rate for pCCA than for dCCA and GBC [11,13,14,34]. The reasons for this are that puncture of pCCA often occurs from the duodenal bulb, making it challenging to stabilize scope manipulation, small lesions or thickened walls are relatively common, and the proximity to the lesion site can be difficult. Obtaining tissue with sufficient strokes in the study participants was difficult, which may have resulted in insufficient tumor-tissue volume and reduced NCCOP suitability.

Ikeda et al. reported that the suitability of NCCOP analysis of EUS-TA specimens in 150 cases of unresectable pancreatic cancer was 39.2%, with 19-gauge and FNB needles being independent factors for a successful analysis [22]. The present study used the same method to assess NCCOP analysis suitability as in this previous report, and EUS-TA was considered useful for patients with UR-BTC (34.9%), as it was for pancreatic cancer (39.2%), with respect to the NCCOP analysis. However, 19-gauge needles and FNB needles did not contribute to NCCOP analysis suitability in this study. The assumed reason for this is that EUS-TA of primary lesions of BTC often has a smaller target than pancreatic cancer. This increases the difficulty of puncture, therefore, 22-gauge needles and FNA needles, which are easier to puncture with, did not result in obtaining relatively fewer tissue samples. Moreover, it is possible that the small number of cases in which 19-gauge needles were used for CGP during EUS-TA may have prevented differences in NCCOP analysis suitability in terms of needle size.

Actionable driver mutations have been reported to be present in 40%–58% of BTC, including iCCA [35,36]. In the present study, all four cases submitted to the NCCOP were successfully analyzed, and actionable mutations were detected. Despite the small number of cases, the results suggest that EUS-TA specimens from BTCs are qualitatively suitable for CGP analysis.

No previous reports of CGP analysis using TPB specimens for BTC are available, and TPB is rarely used for CGP analysis in clinical practice due to the low sensitivity of 49%–67% for malignancy diagnosis in meta-analyses [11,12] and the small size of the specimens obtained. In this study, only 8.6% of participants in the TPB group achieved NCCOP analysis suitability, which is insufficient for real-world clinical use. Comparisons by primary lesion revealed that NCCOP analysis suitability was achieved in a small number of cases of pCCA (10.0%) and dCCA (25.0%). NCCOP analysis suitability of 0% for GBC and iCCA may be mainly due to the fact that the lesion is predominantly located outside the bile duct, for which EUS-TA may be an alternative. In the TPB group, significantly higher NCCOP analysis suitability was observed for the papillary-type lesions and POCS-assisted biopsies. Previous reports have shown that nodular or flat cholangiocarcinoma has a lower sensitivity to malignant diagnosis than papillary type [37]. We speculate that the papillary type of cholangiocarcinoma grows with volume in the bile duct lumen, therefore, there is less contamination of normal tissue during biopsy, allowing us to obtain sufficient tumor tissue for NCCOP analysis. Furthermore, although POCS-compatible biopsy forceps have a small cup, adequate tissue is believed to be obtained by reliably capturing the tumor under direct vision by POCS [38]. Therefore, CGP analysis using TPB specimens may be considered if the lesion is papillary or if there is sufficient tumor volume and good tissue collection under POCS is expected. Furthermore, the rate of meeting the tumor cellularity cutoff of ≥20% in the TPB group in this study was comparable to that of the EUS-TA group (57.1% vs. 58.1%, *p* = 1.000), and the main reason for not meeting NCCOP analysis suitability was tissue area. This suggests that the CGP analysis has the potential to be successful if a sufficient amount of tissue is obtained. Although POCS-compatible biopsy forceps with a larger cup, as used in this study, have been reported as being able to obtain significantly larger amounts of tissue compared to using conventional forceps [39], these are still not sufficient for CGP analysis. The widespread use of larger-channel POCS and the ability to use biopsy forceps with larger cups will enable sufficient tissue to be obtained and contribute significantly to the success of CGP analysis.

Previous reports of EUS-TA for malignant biliary stricture and gallbladder lesions have described adverse event rates of 0–2.0%, examples of which are bleeding and biliary peritonitis [15,34,40,41]. In the present study, the incidence of adverse events in EUS-TA was similar to previously reported rates, and no severe complications were observed. In addition, all patients with pancreatitis in the TPB group underwent not only biopsy but also biliary drainage, making it difficult to assess adverse events deriving from the biopsy itself.

This study has several limitations. First, this was a single-center retrospective study of a small number of cases, which was a major limitation. Second, there was a difference in the proportion of TPB and EUS-TA groups by primary lesions. Third, the primary endpoint was not the actual success rate of the CGP analysis success rate. Fourth, samples of few patients were actually submitted to CGP testing. In the future, additional cases should be collected in multicenter studies to investigate the DNA content of tumor samples directly related to the actual CGP analysis and to validate the results of this study.

## 5. Conclusions

EUS-TA for the primary UR-BTC lesion is an excellent method to obtain suitable tissue specimens for NCCOP analysis, and large lesions with target size ≥ 18 mm may be a good indicator of good target lesions. Conversely, TPB may complement EUS-TA for target lesions with low NCCOP analysis suitability, such as pCCA, if tissue volumes can be obtained.

## Figures and Tables

**Table 1:** Patient characteristics.

Characteristics	All Patients, n = 78	TPB Group, n = 35	EUS-TA Group, n = 43	*p*-Value
Age, years, median (IQR)	71 (64–79)	72 (66–80)	71 (61–77)	0.393
Sex, Male, n (%)	50 (64.1)	27 (77.1)	23 (53.5)	0.035
Primary tumor, n (%)				
pCCA	27 (34.6)	20 (57.1)	7 (16.3)	<0.001
iCCA	20 (25.6)	1 (2.9)	19 (44.2)	<0.001
GBC	17 (21.8)	5 (14.3)	12 (27.9)	0.177
dCCA	7 (9.0)	4 (11.4)	3 (7.0)	0.694
AC	7 (9.0)	5 (14.3)	2 (4.7)	0.233
T category, n (%)				
T4	33 (42.3)	14 (40.0)	19 (44.2)	0.819
T3	40 (51.3)	17 (48.6)	23 (53.5)	0.820
T2	5 (6.4)	4 (11.4)	1 (2.3)	0.168
Form of primary tumor, n (%)				
Mass lesion	56 (71.8)	24 (68.6)	32 (74.4)	0.619
Thickened wall	22 (28.2)	11 (31.4)	11 (25.6)	-
Reason for unresectable, n (%)				
Metastasis	51 (65.4)	18 (51.4)	33 (76.7)	0.031
Locally advanced	21 (26.9)	13 (37.1)	8 (18.6)	0.078
Poor performance status	6 (7.7)	4 (11.4)	2 (4.7)	0.400
Purpose of tissue acquisition, n (%)				
Histological diagnosis	74 (94.9)	35 (100)	39 (90.7)	0.123
CGP	4 (5.1)	0 (0)	4 (8.7)	-
Timing of tissue acquisition, n (%)				
Before chemotherapy	70 (89.7)	34 (97.1)	36 (83.7)	0.068
During chemotherapy	8 (10.3)	1 (2.9)	7 (15.2)	-

TPB, transpapillary biopsy; EUS-TA, endoscopic ultrasound-guided tissue acquisition; IQR, interquartile range; pCCA, perihilar cholangiocarcinoma; iCCA, intrahepatic cholangiocarcinoma; GBC, gallbladder cancer; dCCA, distal cholangiocarcinoma; AC, ampullary cancer; CGP, comprehensive genome profiling.

**Table 2:** Procedural details of biopsy.

Procedural Details	All Patients, n = 78	TPB Group, n = 35	EUS-TA Group, n = 43	*p*-Value
Type of biopsy forceps, n (%)				
POCS-compatible biopsy forceps	20 (25.6)	20 (57.1)	-	-
Standard biopsy forceps	9 (11.5)	9 (25.7)	-	-
One-sided opening-cup biopsy forceps	6 (7.7)	6 (17.1)	-	-
Peroral cholangioscopy, n (%)	4 (5.1)	4 (11.4)	-	-
Needle type, n (%)				
FNB	32 (41.0)	-	32 (74.4)	-
FNA	11 (14.1)	-	11 (25.6)	-
Needle size, n (%)				
22-gauge	35 (44.9)	-	35 (81.4)	-
19-gauge	8 (10.3)	-	8 (18.6)	-
Puncture route, n (%)				
Transduodenal	25 (32.1)	-	25 (58.1)	-
Transgastric	18 (23.1)	-	18 (41.9)	-
Suction method, n (%)				
Slow pull	30 (38.5)	-	30 (69.8)	-
Suction (20 mL)	13 (16.7)	-	13 (30.2)	-
Target size, mm, median (IQR)				
Mass lesion	28.4 (19.8–42.6)	24.5 (18.9–30.4)	34.3 (20.0–46.9)	0.122
Thickened wall	7.0 (5.3–8.2)	6.0 (4.3–8.0)	7.3 (7.0–9.4)	0.263
Number of biopsies, median (IQR)	3 (2–4)	3 (2–4)	3 (2–4)	0.742
Adverse events, n (%)	7 (9.0)	6 (17.1)	1 (2.3)	0.041

TPB, transpapillary biopsy; EUS-TA, endoscopic ultrasound-guided tissue acquisition; POCS, peroral cholangioscopy; FNB, fine-needle biopsy; FNA, fine-needle aspiration; IQR, interquartile range.

**Table 3:** Adequacy of sample in each comprehensive genomic profiling (CGP) analysis suitability criterion.

CGP	Variables	All Patients, n = 78	TPB Group, n = 35	EUS-TA Group, n = 43	*p*-Value
	Cases with tumor cellularity ≥ 20%, n (%)	45 (57.7)	20 (57.1)	25 (58.1)	1.000
	Cases with tissue area ≥ 4 mm^2^, n (%)	23 (29.5)	5 (14.3)	18 (41.9)	0.012
	Cases with tissue area ≥ 25 mm^2^, n (%)	0 (0)	0 (0)	0 (0)	-
NCCOP criteria	Cases with tumor cellularity ≥20% and tissue area ≥ 4 mm^2^, n (%)	18 (23.1)	3 (8.6)	15 (34.9)	0.007
F1CDx criteria	Cases with tumor cellularity ≥ 20% and tissue area ≥ 25 mm^2^, n (%)	0 (0)	0 (0)	0 (0)	-

TPB, transpapillary biopsy; EUS-TA, endoscopic ultrasound-guided tissue acquisition; NCCOP, OncoGuide NCC Oncopanel System; F1CDx, FoundationOne CDx; CGP, comprehensive genomic profiling.

**Table 4:** Univariate and multivariate analyses of factors associated with adequate tissue sampling for OncoGuide NCC Oncopanel System.

Factors	All Patients (n = 78)				
	Adequate, % (n)	Univariate		Multivariate	
		OR (95% CI)	*p*-Value	aOR (95% CI)	*p*-Value
Method of tissue acquisition					
EUS-TA	34.9 (15/43)	5.60 (1.38–33.3)	0.007	5.32 (1.31–21.6)	0.019
TPB	8.6 (3/35)	-	-		
Primary tumor site					
pCCA	7.4 (2/27)	0.18 (0.04–0.83)	0.023	0.33 (0.06–1.79)	0.200
iCCA	35.0 (7/20)	2.30 (0.74–7.11)	0.216		
Others (reference)	29.0 (9/31)	1.0 (reference)	-		
T category					
T4	18.2 (6/33)	0.61 (0.17–2.06)	0.427	-	-
T2 or T3	26.7 (12/45)	-	-		
Form of primary tumor					
Mass lesion	28.6 (16/56)	3.94 (0.80–38.7)	0.080	3.62 (0.71–18.4)	0.121
Thickened wall	9.1 (2/23)	-	-		
Unresectable status					
Metastatic	27.5 (14/51)	2.16 (0.58–10.1)	0.266	-	-
Non-metastatic	14.8 (4/27)	-	-		
Purpose of tissue acquisition					
Histological diagnosis	23.0 (17/74)	-	1.000	-	-
CGP	25.0 (1/4)	1.11 (0.02–15.0)	-		
Timing of tissue acquisition					
Before chemotherapy	22.9 (16/70)	-	1.000	-	-
During chemotherapy	25.0 (2/8)	1.12 (0.10–7.12)	-		
Number of biopsies					
≥3	26.7 (12/45)	1.63 (0.49–6.02)	0.427	-	-
<3	18.2 (6/33)	-	-		

OR, odds ratio; CI, confidence interval; aOR, adjusted odds ratio; EUS-TA, endoscopic ultrasound-guided tissue acquisition; TPB, transpapillary biopsy; pCCA, perihilar cholangiocarcinoma; iCCA, intrahepatic cholangiocarcinoma; CGP, comprehensive genome profiling.

**Table 5:** Univariate analyses of factors associated with adequate tissue sampling for OncoGuide NCC Oncopanel System in the transpapillary biopsy (TPB) group.

Factors	TPB Group (n = 35)	
	Adequate, % (n)	Univariate
		*p*-Value
Primary tumor site		
pCCA/dCCA	12.5 (3/24)	0.536
Others	0 (0/11)	-
T category		
T4	0 (0/14)	0.259
T2 or T3	14.3 (3/21)	-
Form of primary tumor		
Mass lesion	8.3 (2/24)	1.000
Thickened wall	9.1 (1/11)	-
Macroscopic type (exc. iCCA)		
Papillary type	66.7 (2/3)	0.016
Nodular or flat type	3.2 (1/31)	-
Number of biopsies		
≥3	5.0 (1/20)	0.565
<3	13.3 (2/15)	-
Type of biopsy forceps		
POCS compatible	15.0 (3/20)	0.244
Others	0 (0/15)	-
POCS		
Used	50.0 (2/4)	0.029
Not used	3.3 (1/31)	-

pCCA, perihilar cholangiocarcinoma; dCCA, distal cholangiocarcinoma; iCCA, intrahepatic cholangiocarcinoma; POCS, peroral cholangioscopy; TPB, transpapillary biopsy.

**Table 6:** Univariate analyses of factors associated with adequate tissue sampling for OncoGuide NCC Oncopanel System in endoscopic ultrasound-guided tissue acquisition (EUS-TA) group.

Factors	EUS-TA Group (n = 43)	
	Adequate, % (n)	Univariate
		*p*-Value
Primary tumor site		
pCCA	0 (0/7)	0.077
Others	41.7 (15/36)	-
T category		
T4	31.6 (6/19)	0.755
T2 or T3	37.5 (9/24)	-
Form of primary tumor		
Mass lesion	43.8 (14/32)	0.065
Thickened wall	9.1 (1/11)	-
Macroscopic type (exc. iCCA)		
Papillary type	100 (1/1)	0.333
Nodular or flat type	30.4 (7/23)	-
Number of biopsies		
≥3	44.0 (11/25)	0.199
<3	22.2 (4/18)	-
Needle type		
FNB	37.5 (12/32)	0.719
FNA	27.3 (3/11)	-
Needle size		
19-gauge	37.5 (3/8)	1.000
22-gauge	34.3 (12/35)	-
Target size (only mass lesion)		
≥18.5 mm	51.9 (14/27)	0.053
<18.5 mm	0 (0/5)	-

pCCA, perihilar cholangiocarcinoma; iCCA, intrahepatic cholangiocarcinoma; FNB, fine-needle biopsy; FNA, fine-needle aspiration; EUS-TA, endoscopic ultrasound-guided tissue acquisition.

## Data Availability

The authors confirm that the data supporting the findings of this study are available within the article and its Supplementary Materials.

## Supplementary Materials

The following supporting information can be downloaded at: https://www.mdpi.com/article/10.3390/cancers16162819/s1, Figure S1: primary tumor forms and measurement methods in biliary tract cancer; Table S1: proportion of macroscopic type of primary tumor; Table S2: comparison of primary tumor in groups that met the OncoGuide^TM^ NCC Oncopanel System analysis suitability criteria title; Table S3: genetic mutations on OncoGuide^TM^ NCC Oncopanel System analysis.
