# Supplementary material for: Utility of Transpapillary Biopsy and Endoscopic Ultrasound-Guided Tissue Acquisition for Comprehensive Genome Profiling of Unresectable Biliary Tract Cancer
Source: Cancers (Basel). 2024 Aug 10;16(16):2819. doi: 10.3390/cancers16162819 (PMC11353131; doi:10.3390/cancers16162819)
Supplement: Supplementary file 1 [file cancers-16-02819-s001.zip › Figure S1.pdf]

Figure S1. Primary tumor forms and measurement methods in biliary tract cancer.

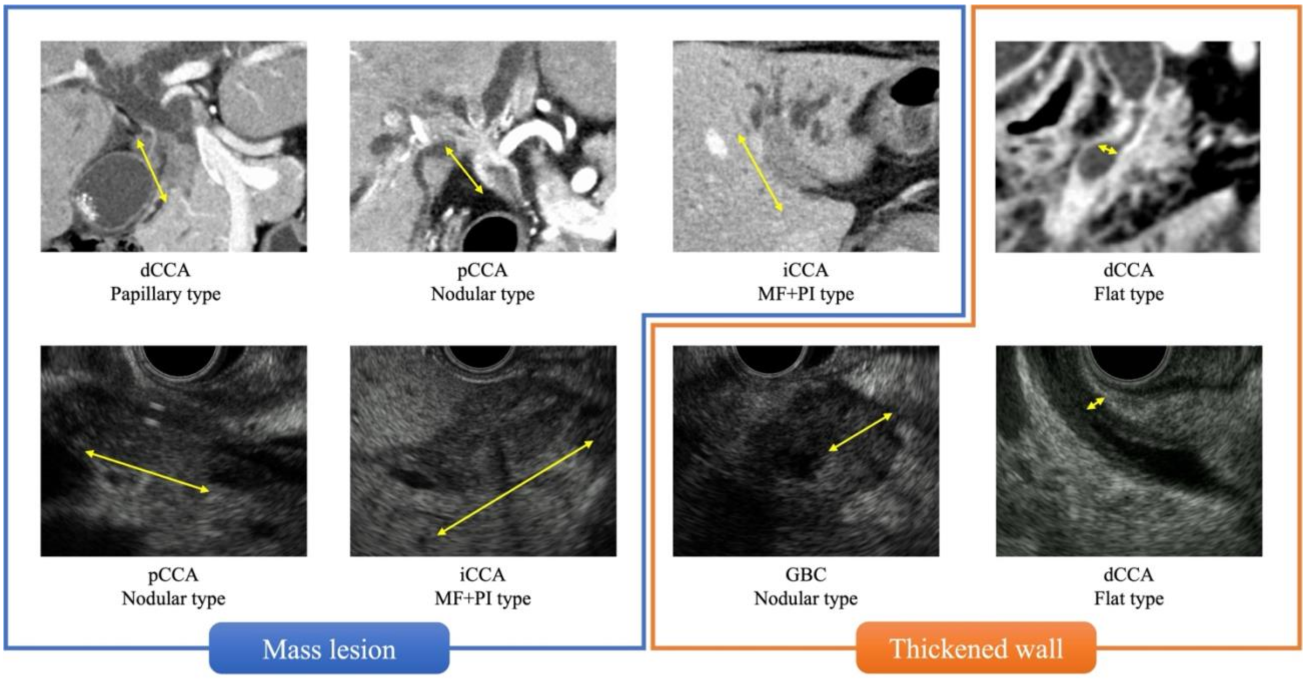

Double-headed arrows indicate tumor size. dCCA, distal cholangiocarcinoma; pCCA, perihilar cholangiocarcinoma; iCCA, intrahepatic cholangiocarcinoma; GBC, gallbladder cancer; MF+PI, mass-forming plus periductal-infiltrating.
