# Supplementary material for: Utility of Transpapillary Biopsy and Endoscopic Ultrasound-Guided Tissue Acquisition for Comprehensive Genome Profiling of Unresectable Biliary Tract Cancer
Source: Cancers (Basel). 2024 Aug 10;16(16):2819. doi: 10.3390/cancers16162819 (PMC11353131; doi:10.3390/cancers16162819)
Supplement: Supplementary file 1 [file cancers-16-02819-s001.zip › Table S1.pdf]

Table S1. Proportion of macroscopic type of primary tumor

Pimary tumor	Macroscopic type	All patients, N=78	TPB group, N=35	EUS-TA group, N=43	p-value
**pCCA, dCCA, GBC, AC, n (%)**	**Overall**	**58 (74.4)**	**34 (97.1)**	**24 (55.8)**	
	- Nodular type	46 (59.0)	25 (71.4)	21 (48.8)	0.324
	- Flat type	8 (10.3)	6 (17.1)	2 (4.7)	0.449
	- Papillary type	4 (5.1)	3 (8.6)	1 (2.3)	0.635
**iCCA, n (%)**	**Overall**	**20 (25.6)**	**1 (2.9)**	**19 (44.2)**	
	- MF+PI type	16 (20.5)	1 (2.9)	15 (34.9)	1.000
	- MF type	4 (5.1)	0 (0)	4 (9.3)	1.000
	- PI type	0 (0)	0 (0)	0 (0)	-
	- IG type	0 (0)	0 (0)	0 (0)	-

TPB, transpapillary biopsy; EUS-TA, endoscopic ultrasound-guided tissue acquisition;
pCCA, perihilar cholangiocarcinoma; dCCA, distal cholangiocarcinoma; GBC, gallbladder cancer;
AC, ampullary cancer; iCCA, intrahepatic cholangiocarcinoma; MF, mass-forming; PI, periductal-infiltrating;
IG, intraductal-growth
