# Supplementary material for: Utility of Transpapillary Biopsy and Endoscopic Ultrasound-Guided Tissue Acquisition for Comprehensive Genome Profiling of Unresectable Biliary Tract Cancer
Source: Cancers (Basel). 2024 Aug 10;16(16):2819. doi: 10.3390/cancers16162819 (PMC11353131; doi:10.3390/cancers16162819)
Supplement: Supplementary file 1 [file cancers-16-02819-s001.zip › Table S3.pdf]

Table S3. Genetic mutations on OncoGuide NCC Oncopanel System analysis

Case	Tumor	Needle	Amount of DNA (ng)	Genetic mutation
1	iCCA	22-gauge FNB	382.7	*IDH1, TP53*
2	GBC	22-gauge FNB	425.7	*GNAQ, SMAD4, TP53*
3	GBC	22-gauge FNB	582.4	*ATM*
4	AC	22-gauge FNB	3731.0	*ARID2, RB1, TP53*

pCCA, perihilar cholangiocarcinoma; iCCA, intrahepatic cholangiocarcinoma; GBC, gallbladder cancer; dCCA, distal cholangiocarcinoma; AC, ampullary cancer; FNB, fine-needle biopsy
