# Supplementary material for: Utility of Transpapillary Biopsy and Endoscopic Ultrasound-Guided Tissue Acquisition for Comprehensive Genome Profiling of Unresectable Biliary Tract Cancer
Source: Cancers (Basel). 2024 Aug 10;16(16):2819. doi: 10.3390/cancers16162819 (PMC11353131; doi:10.3390/cancers16162819)
Supplement: Supplementary file 1 [file cancers-16-02819-s001.zip › Table S2.pdf]

Table S2. Comparison of primary tumor in groups that met the OncoGuide^{TM} NCC Oncopanel System analysis suitability criteria

Primary tumor	All patients, N=78	TPB group, N=35	EUS-TA group, N=43	p-value
iCCA, % (n/N)	35.0 (7/20)	0 (0/1)	36.8 (7/19)	1.000
pCCA, % (n/N)	7.4 (2/27)	10.0 (2/20)	0 (0/7)	1.000
dCCA, % (n/N)	28.6 (2/7)	25.0 (1/4)	33.3 (1/3)	1.000
GBC, % (n/N)	29.4 (5/17)	0 (0/5)	41.7 (5/12)	0.245
AC, % (n/N)	28.6 (2/7)	0 (0/5)	100 (2/2)	0.048

TPB, transpapillary biopsy; EUS-TA, endoscopic ultrasound-guided tissue acquisition; iCCA, intrahepatic cholangiocarcinoma; pCCA, perihilar cholangiocarcinoma; dCCA, distal cholangiocarcinoma; GBC, gallbladder cancer; AC, ampullary cancer
